# From Unicentric Castleman Disease to Lymphoma: A Rare Case Highlighting a Diagnostic and Therapeutic Challenge

**DOI:** 10.7759/cureus.109644

**Published:** 2026-05-25

**Authors:** Nicolás A Jara Rivera, Tabbata D Andrade Castro, Sebastian Vega, Stefanni Vivanco, Angello Muguruza, Rogerio Feijoo

**Affiliations:** 1 Medicine, Universidad Internacional del Ecuador, Quito, ECU; 2 Faculty of Medicine, Universidad de Las Américas, Quito, ECU; 3 Medicine, Universidad Peruana Cayetano Heredia, Lima, PER; 4 Medicine, Universidad Ricardo Palma, Lima, PER; 5 Biology, Pontificia Universidad Católica del Ecuador, Quito, ECU

**Keywords:** case report, castleman disease, hodgkin lymphoma, lymphoma, lymphoproliferative

## Abstract

Unicentric Castleman disease (UCD) is a rare lymphoproliferative disorder treated with surgical resection. However, its association with lymphoma, particularly nodular lymphocyte-predominant Hodgkin lymphoma (NLPHL), is rare and not well characterized. We report a case highlighting the potential for malignant transformation in an otherwise asymptomatic patient. The patient was a 28-year-old male with a history of UCD (hyaline vascular type) in the mediastinum who presented for routine follow-up of previously enlarged lymph nodes. Surveillance chest CT revealed a new posterior mediastinal lymph node. He was asymptomatic, with an unremarkable examination and laboratory studies except for a shortened activated partial thromboplastin time (aPTT). Histopathology showed lymphocyte-predominant (LP) cells with immunophenotypic positivity for CD20, BCL-6, and epithelial membrane antigen (EMA), consistent with NLPHL. Due to the diagnostic complexity, he was treated as having a non-Hodgkin lymphoma-like entity with R-CHOP chemotherapy (rituximab, cyclophosphamide, doxorubicin, vincristine, and prednisone)

Although UCD is typically considered curable with surgical resection, this case demonstrates that, in rare cases, it can progress to lymphoma, specifically NLPHL. The clinical course was asymptomatic, highlighting the variability in presentation and the need for a high index of suspicion in patients with recurrent mediastinal masses. Furthermore, the sequential development of UCD and NLPHL underscores the biological complexity of lymphoproliferative disorders and the potential overlap in immunophenotypic features. This case illustrates that even localized forms of UCD may be associated with subsequent lymphoid malignancy, emphasizing the importance of continuous clinical surveillance and a multidisciplinary approach to management. Histopathological reassessment of new lesions is crucial, and clinicians should consider the possibility of malignant transformation in patients with a history of UCD to ensure accurate diagnosis and timely treatment.

## Introduction

Castleman disease (CD) is a rare, non-clonal lymphoproliferative disorder characterized by heterogeneous clinical presentations and distinct histopathological subtypes. It is broadly classified into unicentric (UCD) and multicentric (MCD) forms based on clinical and anatomical involvement [1]. CD is an uncommon condition, with an estimated incidence of fewer than 25 cases per million person-years, of which approximately 75% are UCD [2,3]. The pathogenesis involves dysregulated immune activation, often driven by excessive cytokine production, particularly interleukin-6 (IL-6). In a subset of cases, most notably MCD, this process is associated with human herpesvirus-8 (HHV-8) infection [4].

Clinically, UCD typically presents as localized lymph node enlargement and is often curable with surgical resection. In contrast, MCD is characterized by systemic inflammation, generalized lymphadenopathy, and a more aggressive clinical course [5]. Although CD is considered a non-malignant disorder, its association with lymphoid malignancies has been increasingly recognized, particularly in patients with MCD [6]. The relationship between UCD and lymphoma, however, remains poorly defined. Rare cases of lymphoma arising in patients with UCD have been reported, suggesting possible shared pathogenic mechanisms, histopathological overlap, or misclassification in select cases [7]. 

We present a rare case of UCD evolving into nodular lymphocyte-predominant Hodgkin lymphoma (NLPHL), an association that is exceptionally uncommon and not well characterized in the literature. This report underscores the potential for malignant transformation even in localized disease and highlights the diagnostic and therapeutic challenges posed by overlapping clinicopathological features.

## Case presentation

A 28-year-old male with a past medical history significant for a left thoracoscopy with biopsy of a mediastinal mass at age 24, which had demonstrated progressive transformation of germinal centers, subsequently underwent a left thoracoscopy with wedge resection of the superior pulmonary lobe months later, which confirmed an initial diagnosis of UCD, hyaline vascular type. He presented for an annual follow-up. Surveillance CT of the chest revealed a newly enlarged lymph node in the posterior mediastinum (Figure 1). Following multidisciplinary evaluation by the Hematology and Cardiothoracic Surgery teams, a biopsy of the newly identified lymph node was recommended.

**Figure 1:**
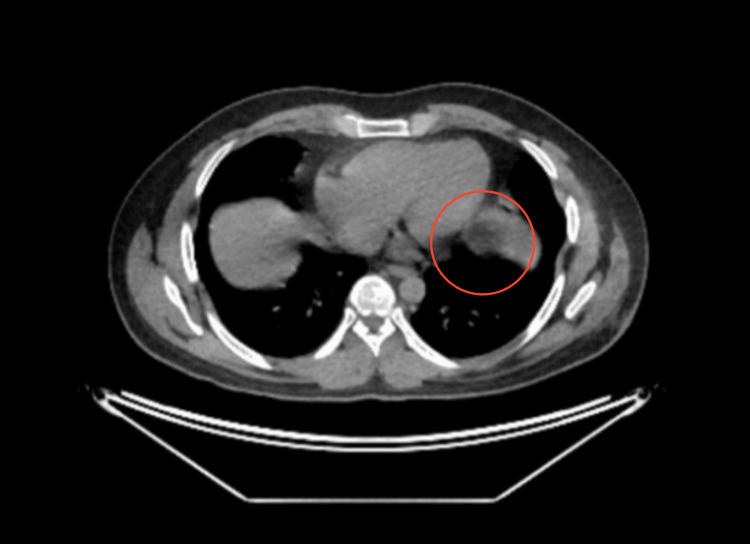
Surveillance CT of the chest Red circle: A hypodense mass is seen in the posterior mediastinum, with surrounding fluid collection in the posterior mediastinal region CT: computed tomography

The patient was admitted for the planned procedure and underwent pre-anesthetic evaluation. On review of systems, he denied chest pain, dyspnea, or other respiratory symptoms. He reported no functional limitations, maintaining his usual level of activity and work capacity. He denied constitutional symptoms, including weight loss or decreased appetite. His medical history was notable for chronic corticosteroid use for an underlying disease. He denied tobacco use and exposure to biomass fuels.

Vital signs were stable, with a blood pressure of 125/86 mmHg, heart rate of 86 bpm, and oxygen saturation of 92% on room air, without clinical signs of respiratory compromise. Physical examination was notable for a large (~20 cm) keloid scar from a prior posterolateral thoracotomy, as well as additional keloid scars at the seventh and eighth intercostal spaces and the right iliac fossa. The remainder of the examination, including the cardiovascular, respiratory, abdominal, and neurological systems, was unremarkable. The patient was alert and oriented to person, place, and time.

Laboratory evaluation, including a complete blood count, coagulation panel, basic metabolic panel, and electrolytes, was performed. The only abnormal finding was a shortened activated partial thromboplastin time (aPTT) (Table 1).

**Table 1:** Complete coagulation panel

Variable	Result	Unit	Reference range
Prothrombin time (PT)	11.9	Seconds	10.0–14.6
International normalized ratio (INR)	0.99	-	≤ 1.3
Prothrombin activity	120.7	%	-
Activated partial thromboplastin time (aPTT)	23.4	Seconds	25.0–40.0

A biopsy was performed via video-assisted thoracoscopic surgery (VATS). On postoperative day (POD) one, the patient demonstrated poor compliance with respiratory exercises. A chest radiograph (CXR) revealed bibasilar atelectasis and a moderate pleural effusion. A chest tube was placed, yielding serosanguineous fluid. Supportive management included supplemental oxygen, initiation of a loop diuretic, and pharmacologic thromboprophylaxis.

On postoperative day three, a repeat CXR demonstrated a significant interval reduction in the pleural effusion; however, a new left apical pneumothorax was identified. Histopathological examination of the mediastinal lymph node revealed a vaguely nodular architecture with an expansive growth pattern, predominantly composed of small lymphocytes surrounding scattered large atypical cells. These cells exhibited multilobulated to round nuclei, delicate nuclear membranes, finely granular chromatin, and variably prominent nucleoli, consistent with lymphocyte-predominant (LP) cells (Figures 2, 3).

**Figure 2:**
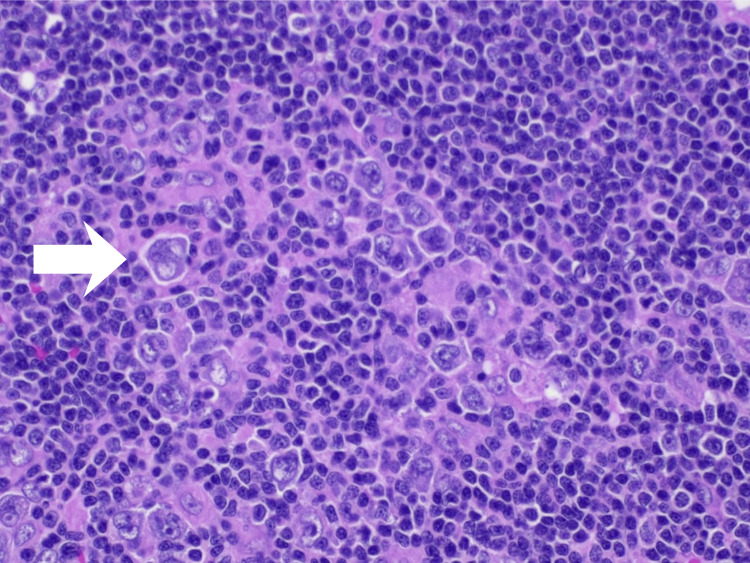
Histopathological examination - image 1 White arrow: a large neoplastic cell with a multilobulated nucleus, delicate nuclear membranes, finely granular chromatin, and nucleoli of variable size, surrounded by small lymphocytes

**Figure 3:**
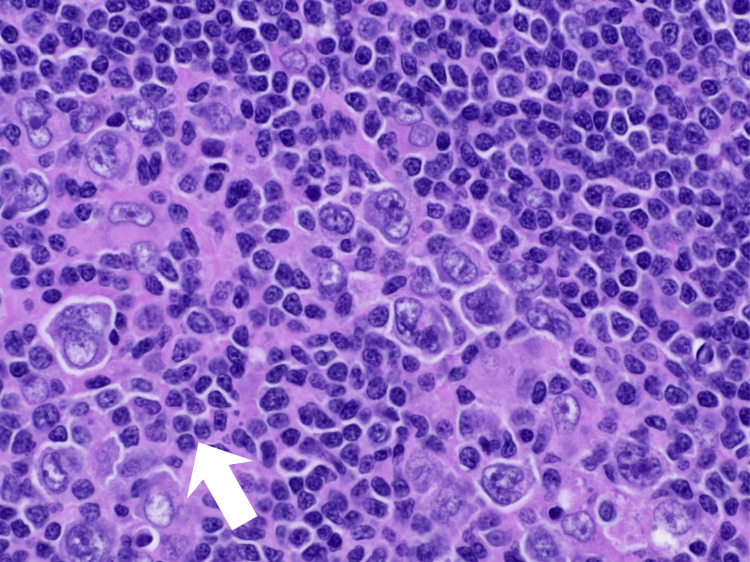
Histopathological examination - image 2 White arrow: small lymphocytes surrounding a large neoplastic cell. The background lymphoid population showed a mixed distribution of CD3/CD4-positive T cells and CD20-positive B cells

Immunophenotypic analysis demonstrated that the neoplastic cells were positive for CD20, BCL-6, and epithelial membrane antigen (EMA), with rare CD15-positive cells. Germinal centers exhibited abnormal morphology with irregular distribution among nodules, although focal preservation of architecture was observed in peripheral regions. Classic Reed-Sternberg cells were not identified; however, some LP cells demonstrated partial morphological resemblance. These findings were consistent with a diagnosis of NLPHL. Given the diagnostic complexity, the patient was referred to a tertiary care center in the United States for further evaluation. Histopathological specimens were re-reviewed, and, in the context of the overall cellular and immunophenotypic profile, a multidisciplinary team, coordinating with the treating physicians in Ecuador, opted to manage the disease as a non-Hodgkin lymphoma-like entity.

Given the diagnostic complexity, overlapping histopathological features, and multidisciplinary consensus favoring a biologically active disease with potential for progression, a decision was made to treat the patient with a systemic chemoimmunotherapy regimen consistent with a non-Hodgkin lymphoma-like approach. The patient was subsequently initiated on R-CHOP chemotherapy (rituximab, cyclophosphamide, doxorubicin, vincristine, and prednisone). The patient tolerated treatment well without significant adverse events. At follow-up, he demonstrated clinical improvement with resolution of postoperative respiratory complications and no new symptoms. Interval imaging showed no evidence of disease progression, and findings were consistent with a favorable response to therapy. He remains under close outpatient surveillance with the Oncology team for continued monitoring.

## Discussion

This report describes a 28-year-old male with a history of unicentric hyaline vascular CD who developed a recurrent mediastinal mass, which was ultimately diagnosed as NLPHL. Although the patient initially followed an expected course with surgically managed recurrences, subsequent histopathological findings revealed a distinct, more aggressive pathology, underscoring the need to reconsider the diagnosis in patients with recurrent lesions despite prior benign results. Similar cases have been reported in the literature, including patients with CD later diagnosed with classical Hodgkin lymphoma presenting with Reed-Sternberg cells and B symptoms, such as fever, weight loss, and night sweats [8]. In contrast, our patient developed NLPHL and remained asymptomatic, highlighting the heterogeneity in clinical presentation and disease evolution.

CD is a heterogeneous condition, with the unicentric hyaline vascular subtype generally considered benign and effectively treated with surgical resection [9]. Malignant transformation or association with lymphoma has been more frequently described in MCD, particularly in HHV-8 associated cases [10]. However, progression from UCD to lymphoma is rare [11]. When lymphoma does occur, it is more commonly associated with non-Hodgkin lymphoma rather than Hodgkin lymphoma [12]. NLPHL itself is an uncommon subtype of Hodgkin lymphoma, characterized by an indolent clinical course and distinct immunophenotypic features [13]. The coexistence or sequential development of CD and NLPHL is rarely reported, making this case an unusual presentation that contributes to the existing literature.

This report emphasizes several important clinical considerations. First, recurrent mediastinal masses in patients with a history of CD should not automatically be assumed to represent benign recurrence; malignancy, including lymphoma, must be considered in the differential diagnosis. Second, imaging findings alone are insufficient to establish the nature of recurrent lesions, and histopathological confirmation remains essential, particularly in cases with atypical evolution or repeated recurrence. Third, this case underscores the importance of multidisciplinary evaluation, including close collaboration between surgeons, pathologists, and oncologists, especially when diagnostic uncertainty exists. The malignant transformation of UCD into lymphoma is a rare event, with only isolated cases reported in the literature [14], further supporting the clinical relevance of maintaining a high index of suspicion in similar scenarios. Additionally, from a surgical standpoint, prior thoracic interventions can significantly increase operative complexity due to adhesions and tissue fragility, as observed in this patient.

The underlying mechanism linking CD and lymphoma remains unclear. One proposed explanation is chronic immune stimulation leading to lymphoid proliferation and eventual malignant transformation [10]. The pathophysiological basis may involve a dysregulated immune microenvironment characteristic of UCD, typically driven by localized lymph node hyperplasia and increased production of IL-6, which promotes B cell proliferation, plasmacytosis, and vascular proliferation [15]. Unlike MCD, this process is usually localized and not associated with systemic inflammation or viral triggers such as HHV-8 [16].

In our patient, the absence of HHV-8 expression and systemic symptoms suggests a localized cytokine-driven process without significant systemic involvement. Additionally, chronic antigenic stimulation and follicular dendritic cell dysregulation may contribute to altered lymphoid architecture and reactive changes that overlap with lymphoproliferative disorders [10]. Also, the diagnostic overlap between NLPHL and other B-cell lymphomas, particularly T-cell/histiocyte-rich large B-cell lymphoma, reflects shared biological features and supports the concept of a spectrum of lymphoproliferative disorders, as recognized in modern classifications [17].

This report has several limitations. As a report involving a single case, it does not establish a causal relationship between CD and the development of NLPHL, but rather suggests a possible association. Additionally, the lack of long-term follow-up limits the ability to assess disease progression, response to therapy, and overall prognosis. Importantly, the absence of earlier pathological reports and incomplete external medical records limit the ability to fully characterize disease evolution. Furthermore, uncertainties regarding prior diagnostic evaluations and the rationale for therapeutic decisions, including the use of non-Hodgkin lymphoma-directed treatment, represent additional limitations. Finally, given the known diagnostic overlap between NLPHL and other lymphomas, clinicopathological correlation is essential, and misclassification cannot be entirely excluded.

Overall, our findings suggest that recurrent mediastinal lesions in patients with prior UCD warrant thorough reevaluation, including repeat biopsy, given that malignant transformation or lymphoma development, although rare, remains possible.

## Conclusions

This report illustrates the uncommon but significant association between UCD and NLPHL. It highlights the need for continued clinical surveillance in UCD patients and calls for further research into the mechanisms behind malignant transformation and optimal management strategies. Clinically, this report adds to the limited evidence linking UCD with lymphomagenesis, particularly NLPHL, and highlights the heterogeneity and potential evolution of lymphoproliferative disorders. It also emphasizes the need for close long-term clinical surveillance in these patients. A high level of clinical suspicion should be maintained, and histopathological confirmation is recommended in recurrent lesions, as imaging alone is insufficient to exclude malignancy. Further studies are needed to elucidate the underlying pathophysiological mechanisms of this association and to establish optimal surveillance and therapeutic strategies.
